# Hemoglobin as a prognostic marker for neurological outcomes in post-cardiac arrest patients: a meta-analysis

**DOI:** 10.1038/s41598-023-45818-5

**Published:** 2023-10-28

**Authors:** Hongxiang Hou, Li Pang, Liang Zhao, Zuolong Liu, Ji-Hong Xing

**Affiliations:** 1 Department of Emergency, the First Hospital of Jilin University, Xinmin Street 1, Chaoyang District, Changchun, China; 2 Rehabilitation Department, the First Hospital of Jilin University, Changchun, China

**Keywords:** Neuroscience, Biomarkers, Diseases, Neurology, Pathogenesis, Risk factors

## Abstract

The aim of this study was to investigate the relationship between serum level of hemoglobin and neurological outcomes following cardiac arrest. Relevant studies were identified by searching electronic databases including PubMed, Web of Science, Cochrane Library, and Embase from June 2012 through April 2023. Articles were rigorously reviewed for their study inclusion and exclusion criteria. Pooled effect date was determined using the standardized mean difference (SMD) and 95% confidence intervals (CI). The Newcastle–Ottawa Scale was used to evaluate study quality. Subgroup analyses were conducted to determine confounding factors affecting patient outcomes. Study heterogeneity, sensitivity, and publication bias were also determined.

This meta-analysis included 11 studies involving 2519 patients. Our results suggest that high serum level of hemoglobin may improve neurological prognosis(SMD = 0.60, 95%CI = 0.49–0.71, *I*^2^ = 10.85). The findings of this study indicate that serum level of hemoglobin may be associated with better neurological prognosis, perhaps an appropriate increase in serum haemoglobin levels can improve the neurological prognosis of patients in cardiac arrest.

## Introduction

Cardiac arrest (CA), including in-hospital cardiac arrest (IHCA) and out-of-hospital cardiac arrest (OHCA), is a significant public health issue. In the United States, more than 600,000 people suffer from cardiac arrest each year^1, 2^, and the incidence of cardiac arrest has increased over recent years^3^. Patients who have suffered from cardiac arrest have high mortality attributed to ischemia hypoxia and reperfusion injury, and those who survive often present with long-term neurological dysfunction and significantly poor prognosis^4^. Therefore, early screening for neurological symptoms should be carried out promptly in patients with cardiac arrest.

Relevant guidelines indicate a multimodal neuro-prognostication strategy to predict neurological outcomes, including measuring blood levels of neuron-specific enolase^5^. NSE is a biomarker that is part of the multimodal evaluation of the post-resuscitation neurological prognosis, recognized in the resuscitation guidelines, but not routinely tested on admission. Hemoglobin, which primarily consists of erythrocytes circulating in the blood, is a significant oxygen transport protein^6^. It has been reported that serum level of hemoglobin correlated with poor neurologic outcomes^7^. Given that serum level of hemoglobin testing is simple and inexpensive, we conducted a meta-analysis to determine the impact of serum level of hemoglobin as a prognostic blood biomarker for neurological outcomes in post-cardiac arrest patients.

## Methods

This study was carried out according to the principles outlined in the A 24-step guide on how to design, conduct, and successfully publish a systematic review and meta-analysis in medical research^8^ and the Preferred Reporting Items for Systematic Reviews and Meta-analysis (PRISMA) guidelines^9^. Our research question was designed considering study population, intervention, comparison, and outcome (PICO) as follows: population (P) = post-cardiac arrest adult patients; intervention (I) = serum Hb level; comparator (C) = none; outcome (O) = neurological outcomes.

### Search strategy

All comprehensive articles published before April 2023 that estimated the neurological prognostic effect of Serum level of hemoglobin in adult patients with cardiac arrest were searched in PubMed, Web of Science, Cochrane Library, and Embase databases by two experienced reviewers (Hong-xiang Hou and Liang Zhao). The reference lists of eligible studies were also searched to identify any studies that were not identified in the initial search.

The following search terms were used: “arrest, heart” or “cardiac arrest” or “arrest, cardiac” or “asystole” or “asystoles” or “cardiopulmonary arrest” or “arrest, cardiopulmonary” or “Ventricular Fibrillation” or “Fibrillation, Ventricular” or “Fibrillations, Ventricular” or “Ventricular Fibrillations” or “Tachycardia” or “Tachycardias or “Tachyarrhythmia” or “Tachyarrhythmias” or “Death, Sudden, Cardiac” or “Sudden Cardiac Death” or “Cardiac Death, Sudden” or “Death, Sudden Cardiac” or “Cardiac Sudden Death” or “Death, Cardiac Sudden” or “Sudden Death, Cardiac” or “Sudden Cardiac Arrest” or “Arrest, Sudden Cardiac” or “Cardiac Arrests, Sudden” or “Cardiac Arrest, Sudden” and “hemoglobin” or “Eryhem” or “ferrous, hemoglobin” or “hemoglobin, ferrous” and “prognoses” or “prognostic factors” or “factor, prognostic”. Study retrieval is shown in Supplementary Table 1.

Two authors carefully reviewed the title and abstract of all articles and independently scanned all articles based on predefined inclusion and exclusion criteria.

Inclusion criteria: (1) the included population was over 18 years of age; (2) non-traumatic cardiac arrest; (3) serum level of hemoglobin were detected; and (4) articles using the cerebral performance categories (CPC) scale to evaluate neurological outcomes.

Exclusion criteria: (1) age < 18 years; (2) patients with traumatic cardiac arrest; (3) intracranial bleeding; and (4) irrelevant topic, letters, duplicate data or publications, reviews, meta-analyses, case reports, comments, editorials, or animal experiments.

### Quality assessment

Two reviewers independently used the Newcastle–Ottawa Scale (NOS) for non-randomized studies to assess the quality of the included studies^10^.The NOS consists of eight items that were divided into three domains: cohort selection, comparability, and outcome assessment. Grading the quality of evidence and strength of recommendations using the GRADE (Grading of Recommendations Assessment, Development and Evaluation) method based on risk of bias, inconsistency, indirectness, imprecision, and publication bias^11, 12^. At the same time, we use the Quality in Prognostic Studies (QUIPS) tool^13^ to assess the risk of bias. The tool consists of six domains: study participation, study attrition, prognostic factor measurement, outcome measurement, study confounding, statistical analysis and reporting. A low, moderate or high risk of bias was assessed for each domain. The original study was reevaluated by a third author when differences arose between the primary reviewers.

### Data extraction

Two independent researchers extracted the relevant data for patients from all eligible studies. Any discordant assessments were resolved by a third investigator. The extracted variables were as follows: first author^’^s name, year of publication, country in which the study was conducted, geographic location, inclusion period, study type, sample size, cardiac arrest type (OHCA vs. IHCA), poor neurologic outcome (PNO), serum hemoglobin sampling time, assessment tool of outcome measurement, point of outcome measurement, mean ± standard deviation (± SD), Hb level, age, male, and CPR duration. If the mean ± SD was not available, interquartile range and median^14–16^ were converted into mean ± SD, using the method established by Wan et al.^17^. Based on the CPC score, neurological outcomes were defined as good or poor.

### Statistical analysis

The association between serum level of hemoglobin and neurological outcome was estimated for every study using the standardized mean difference (SMD) and 95% confidence interval (CI). Serum hemoglobin unit measurements differed between studies. We tried to convert it into uniform units, but we could not achieve it because only the mean value was provided in the article, so we chose to use SMD. The Cochrane Q test (*p* < 0.10) and the *I*^2^ statistic were used to assess heterogeneity among the included studies. The star chart also was used to test heterogeneity. The heterogeneity results of PQ < 0.1 and/or *I*^2^ > 50% were considered high heterogeneity using the random effects models, otherwise PQ ≥ 0.1 and/or *I*^2^ ≤ 50% were considered as statistically significant heterogeneity using the fixed-effects model^18, 19^. A funnel plot and Egger^’^s linear regression test were used to assess publication bias. Sensitivity analysis was used to judge robustness. Stata version 16.0 was used to perform all analyses.

## Results

### Study selection

**Figure 1:**
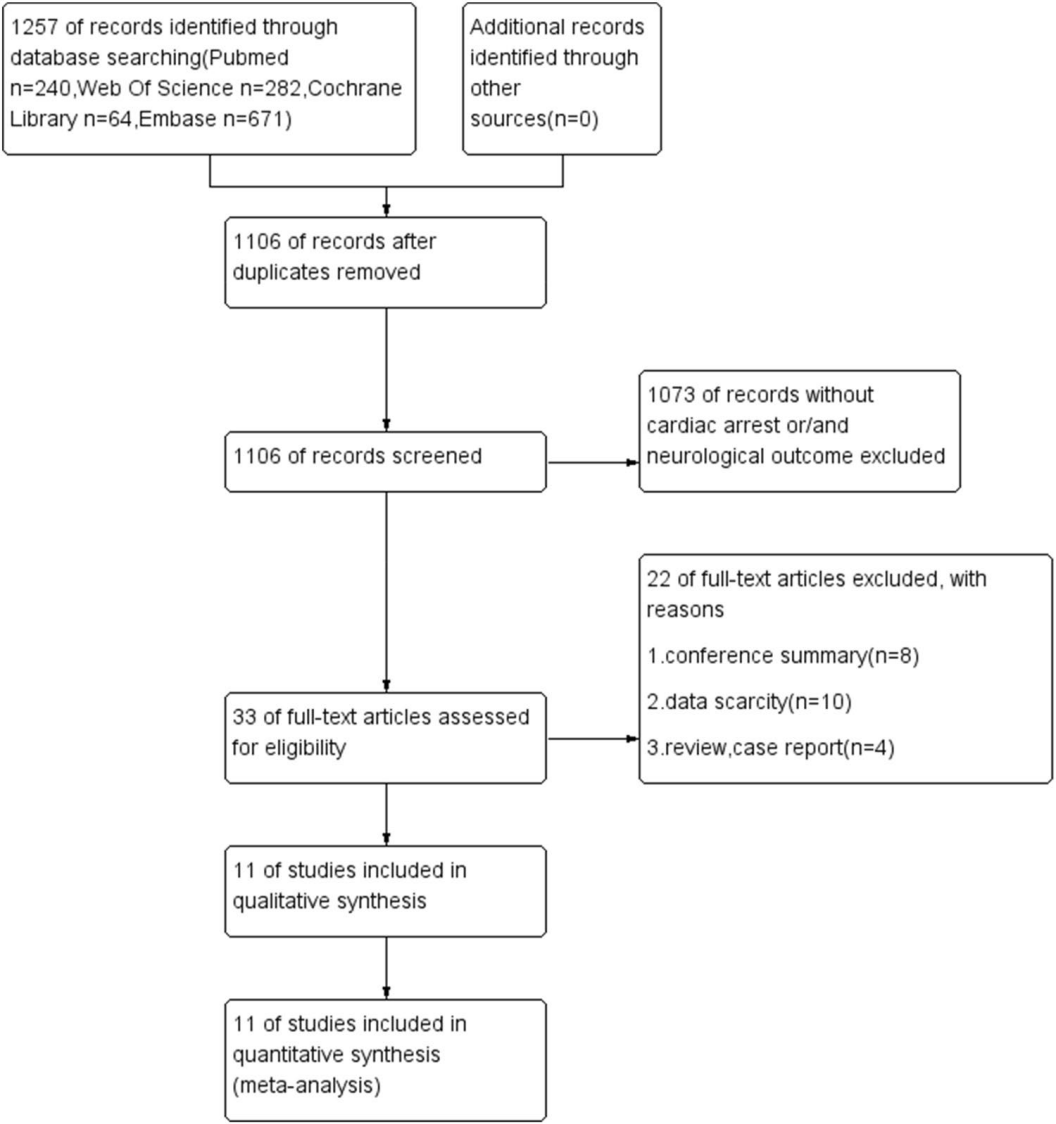
Flow diagram of studies included in the meta-analysis.

Using the established study retrieval method, 1257 studies were identified in the database search: 151 studies were duplicates and 1073 irrelevant publications without cardiac arrest or/and neurological outcomes were excluded after reviewing titles and abstracts. After full-text articles were assessed for eligibility, 11 publications were included in the meta-analysis^7, 14–16, 20–26^. The screening process is shown in Fig. 1.

### Document quality assessment and data extraction

Two studies were multi-center prospective observational studies (mPOS), and one study was a single-center prospective observational study (sPOS). The remaining eight studies were single-center retrospective observational studies (sROS). Seven studies only included OHCA patients, one study only included IHCA patients, and two studies included both OHCA and IHCA patients. One study did not clarify the CA type. All articles used CPC scores for neurologic outcomes. The baseline information of the included studies are presented in Table 1 and Supplementary Table 2. According to NOS analysis, quality scores ranged from 4 to 9, and five studies were rated as high quality. The GRADE protocol was used to assess the certainty of the evidence. The evidence for hemoglobin as a prognostic marker was rated as very low due to risk of bias or evidence of publication bias (Supplementary Fig. 11). QUIPS has been used for the evaluation of prognostic studies. The overall results of the quality assessment are shown in Supplementary Fig. 12.

**Table 1:** Study characteristics.

Author	Year	Country	Race	Geographic location	Inclusion period	Study type	Sample size, CA type	PNO,n (%)	hemoglobin sampling time	Outcome measurement		NOS
										Assessment tool	Time point	
SOS-KANTOstudy group	2012	Japan	Yellow	Asia	2002–2003	mPOS	137,OHCA	103(75)	hospital arrival	CPC(1–2)34 CPC(3–5)103	1 month	5
Aiham Albaeni	2016	USA	White	North America	2004–2010	sROS	146,OHCA	116(79)	ROSC	CPC(1–2)30 CPC(3–5)116	–	4
K. Ameloot	2015	Belgium	White	Europe	–	sROS	82,CA	39	admission	CPC(1–2)7/21(33%) CPC(3–5)36/61(59%)	6 month	5
Federica Zama Cavicchi	2020	Belgium	White	Europe	2007–2015	sRos	414,OHCA231 + IHCA	257	admission	CPC(1–2)157 CPC(3–5)257	3 month	7
Andrew Wormsbecker	2017	Canada	White	North America	2009–2014	sRos	118,OHCA + IHCA	86	Average hemoglobin	CPC(1–2)32 CPC(3–5)86	Hospital discharge	7
Chih-Hung Wang	2016	China	Yellow	Asia	2006–2012	sRos	426IHCA	372	Average hemoglobin	CPC(1–2)54 CPC(3–5)372	Hospital discharge	5
Se Jong Oh	2018	Korea	Yellow	Asia	2007–2013	sROS	295OHCA	216	hospital arrival	CPC(1–2)79 CPC(3–5)216	6 month	5
Young Mo Cho MD	2012	Korea	Yellow	Asia	2007–2010	sROS	117OHCA	83	hospital arrival	CPC (1–2) 34 CPC(3–5)83	1 month	6
Kei Hayashida	2014	Japan	Yellow	Asia	2011–2013	mPOS	495OHCA	420	hospital arrival	CPC (1–2) 75 CPC(3–5)420	3 month	9
Adrien Bouglé	2016	France	White	Europe	2012–2013	sPOS	43OHCA	21	Average hemoglobin	CPC (1–2) 22 CPC(3–5)21	Hospital discharge	7
Daesung Kim	2018	Korea	Yellow	Asia	2009–2015	sROS	246OHCA	159	ROSC	CPC (1–2) 87 CPC(3–5)159	Hospital discharge	5

CA: cardiac arrest, PNO: poor neurologic outcome, OHCA: out-of-hospital cardiac arrest, h: hour, IHCA: in-hospital cardiac arrest, CPC: cerebral performance categories, sPOS: single-center prospective observational study, sROS: single-center retrospective observational study, mPOS: multi-center prospective observational study, ROSC: recovery of spontaneous circulation, NOS: Newcastle–Ottawa Scale.

### Meta-analysis

**Figure 2:**
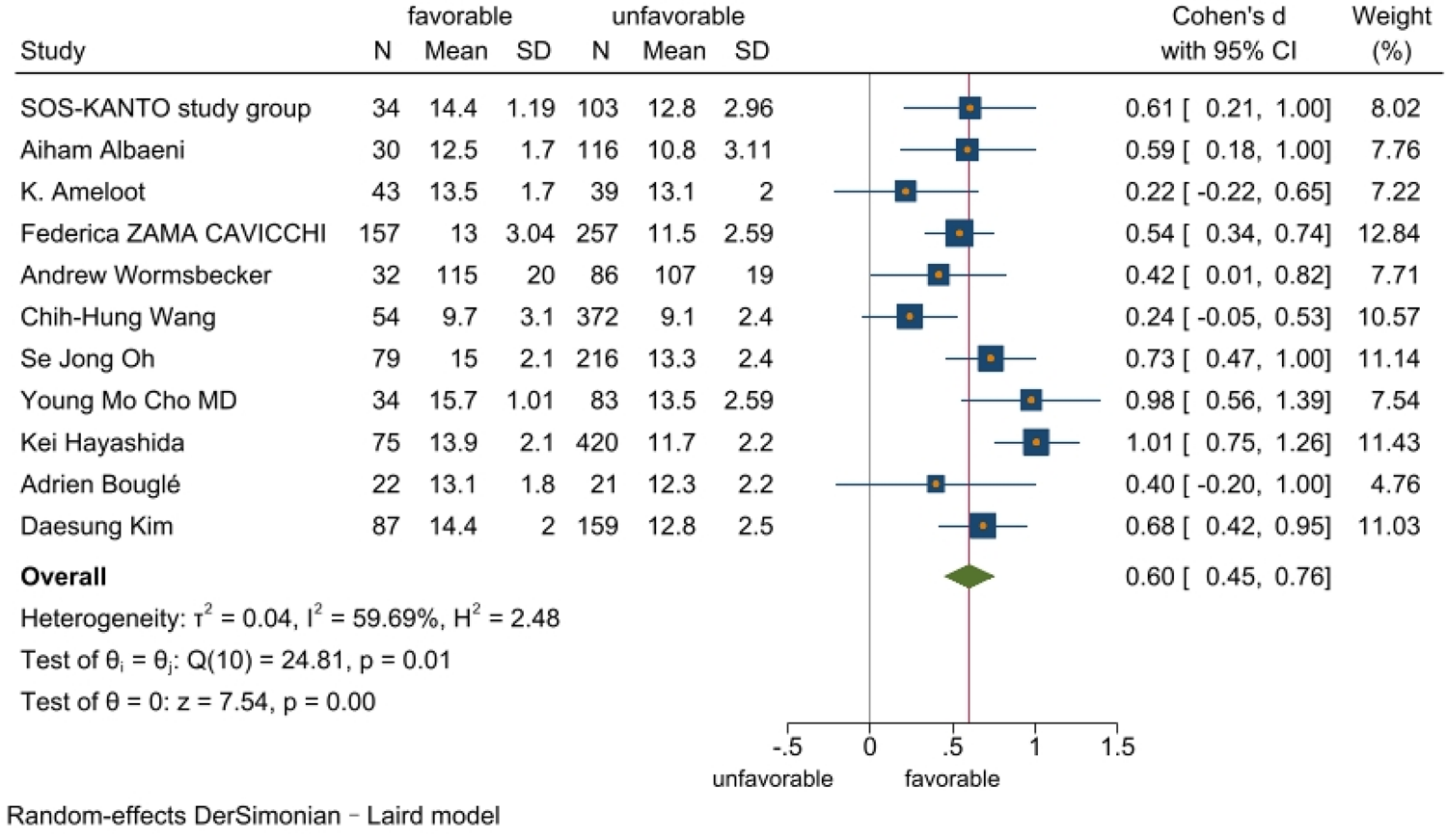
Forest plot of 11 studies. favorable: good neurological prognosis group; unfavorable: poor neurological prognosis group.

**Figure 3:**
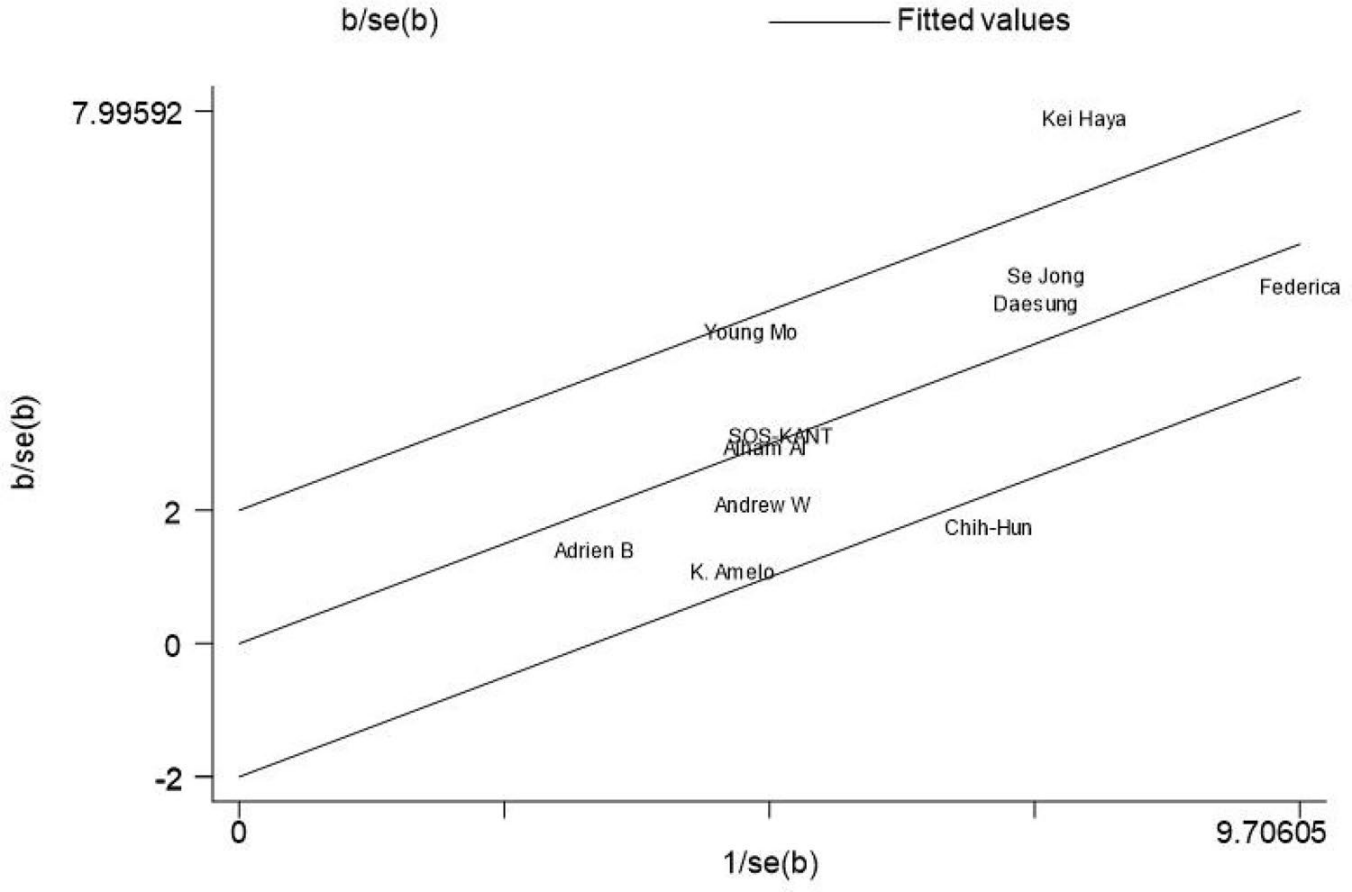
Star chart.

**Figure 4:**
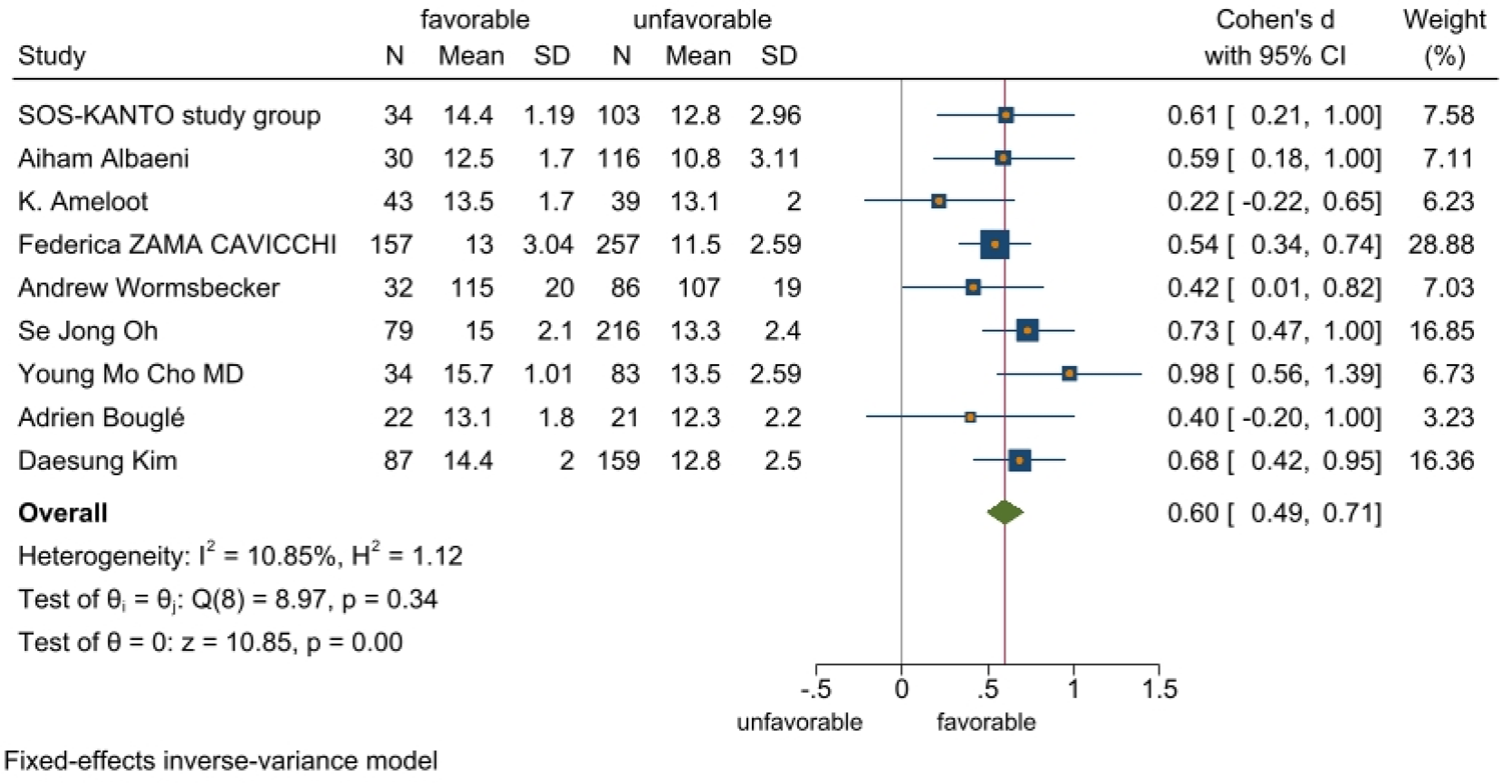
Relationship between serum level of hemoglobin and neurological outcomes. favorable: good neurological prognosis group; unfavorable: poor neurological prognosis group.

**Figure 5:**
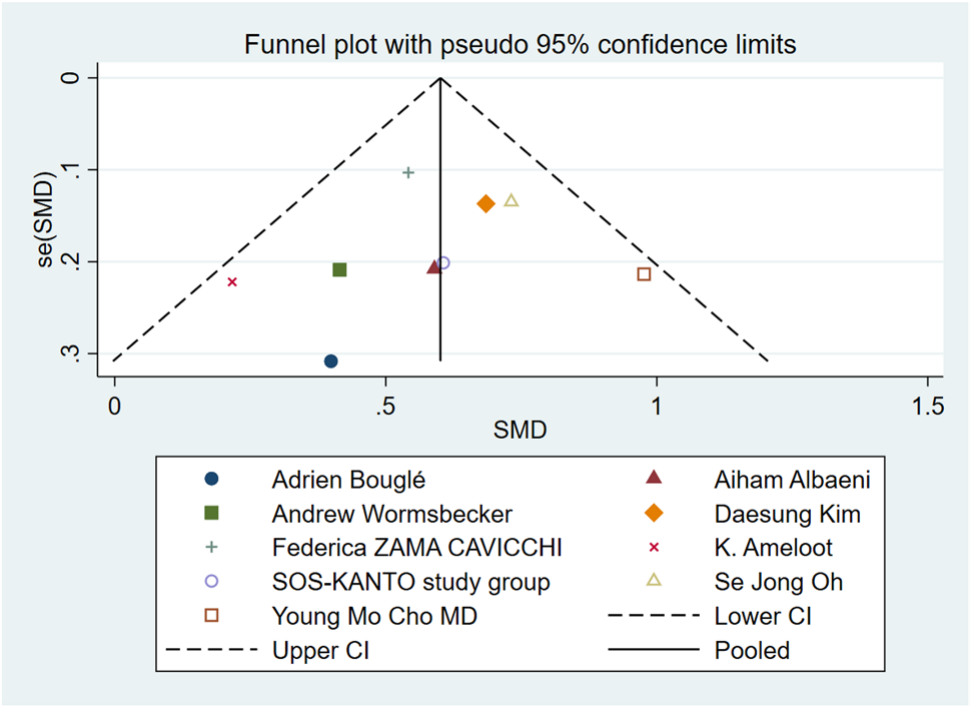
Funnel plot.

**Figure 6:**
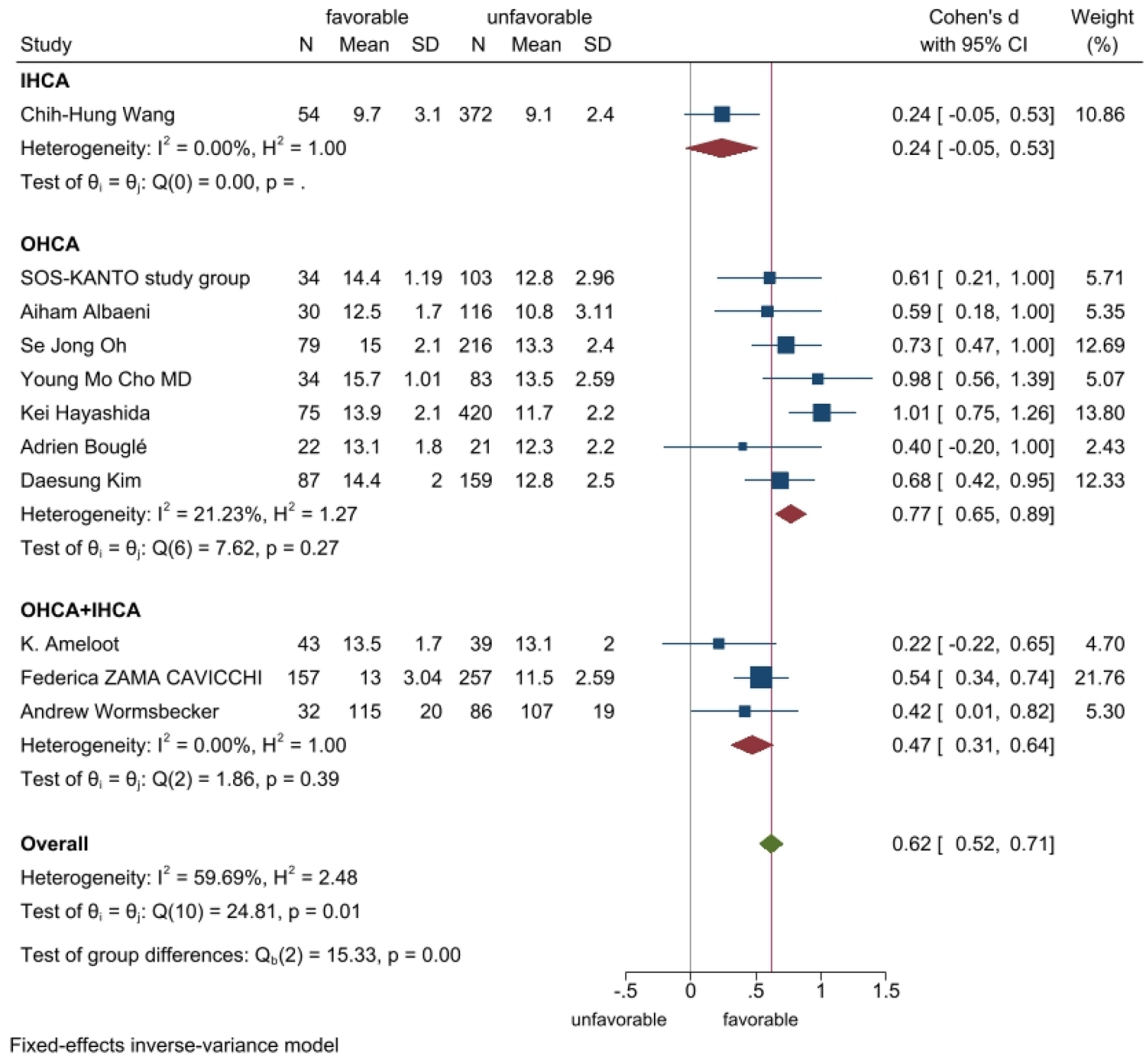
Subgroup analysis. favorable: good neurological prognosis group; unfavorable: poor neurological prognosis group OHCA: out-of-hospital cardiac arrest; IHCA: in-hospital cardiac arrest.

The meta-analysis included 11 studies with a total of 2519 cardiac arrest patients including 647 patients with a good neurological prognosis and 1872 patients with a poor neurological prognosis. Articles in this study had heterogeneity values of *I*^2^ = 59.7% and Q test *P* < 0.1, suggesting that there was heterogeneity among the literatures selected for this study. The random effect model was applied to our meta-analysis (Fig. 2). This analysis showed that serum level of hemoglobin in the good prognosis group was 0.60 higher than that in the poor prognosis group, and this difference was statistically significant (*P* < 0.05). A star chart was used to further investigate heterogeneity (Fig. 3). This analysis indicated the studies by Kei Hayashida et al. and Chih-Hung Wang et al. affected the heterogeneity; removing these two articles from the analysis using the random effect model improved the heterogeneity (Fig. 4). Nine studies with a total of 1598 cardiac arrest patients including a total of 518 patients with good neurological prognosis and 1080 patients with poor neurological prognosis. The SMD value of the 1589 patients was 0.6, with a 95%CI of 0.49–0.71 (Z = 10.85, *P* < 0.05), suggesting that higher serum level of hemoglobin may be a relevant factor for better neurological prognosis. Sensitivity analysis was conducted on the remaining nine articles (Supplementary Fig. 1). Figure 5 shows that the funnel plot analysis was symmetrical with a *P*-value > 5 after testing for publication bias. The Begg’s and Egger’s tests (Supplementary Fig. 2) further suggest there was no publication bias in the nine studies. In the regression analysis (Supplementary Fig. 3), Cardiac arrest type was considered the source of heterogeneity. Therefore, subgroup analysis was conducted according to cardiac arrest type (Fig. 6). We found that cardiac arrest type affected the results of the meta-analysis. The results of other subgroup analysis regarding race, location, research type, serum hemoglobin sampling time, and outcome measurement time point are provided in Supplementary Figs. 4–7. The results indicate that serum hemoglobin sampling time was a source of heterogeneity. We also analysed the relationship between bystander CPR, initial shockable rhythm, time to ROSC and neurological prognosis for which data were available(Supplementary Figs. 8–10). Between Initial shockable rhythm, time to ROSC and neurological prognosis had heterogeneity. Bystander CPR may improve neurological prognosis in patients survived after cardiac arrest.

## Discussion

Hemoglobin is key factor of blood oxygen-carrying capacity, and as such serum hemoglobin correlates with the prognosis of various diseases. There is accumulating evidence demonstrating that serum hemoglobin concentration is associated with poor neurological outcome in a variety of brain injuries^27^, including traumatic brain injury (TBI), subarachnoid hemorrhage (SAH), stroke, and hemorrhage^28–31^. A meta-analysis demonstrated that serum hemoglobin concentration may be associated with mortality after transcatheter aortic valve implantation^32^. A retrospective study demonstrated that serum albumin and serum level of hemoglobin at admission predict mortality in children with TBI^33^. A previous study has shown that higher serum level of hemoglobin at admission were involved in better outcomes in patients with spontaneous, nontraumatic intracerebral hemorrhage^34^ The literature indicates that risk of progression of IgA nephropathy decreases with increases in serum level of hemoglobin^35^. Further evidence suggests that a decline in serum level of hemoglobin by ≥ 3 g/dl is related to an increased risk of mortality in patients with acute coronary syndromes^36^. An earlier study demonstrated that elevating serum hemoglobin concentration at admission may reduce the risk of death after discharge in patients with acute exacerbation of chronic obstructive pulmonary disease^37^. It has been shown that anemic mice have a worse prognosis after traumatic brain injury compared to non-anemic mice, the exact mechanism of which is unclear^38^. A meta-analysis by Mori and colleagues suggested that serum level of hemoglobin were associated with overall mortality and disease progression in patients with metastatic hormone-sensitive prostate cancer^39^. Another meta-analysis revealed that stroke patients who present with anemia from the onset have a higher risk of mortality^40^. A recent study has shown that lower serum level of hemoglobin at admission may predict extent of kidney damage in patients with type 2 diabetes^41^. To the best of our knowledge, our meta-analysis is the first to investigate the association of serum level of hemoglobin with neurological outcomes in patients following cardiac arrest. This meta-analysis showed that higher serum hemoglobin levels may improve neurological prognosis in patients who have survived after cardiac arrest. Our findings are consistent with those of Cavicchi et al.^15^. It was also reported that patients following OHCA who had higher serum level of hemoglobin achieved full neurological recovery^42^. However, because the data was incomplete in that study it was not selected for inclusion in the current meta-analysis^42^ Another study reported that anemia is a risk factor for cardiac arrest, which was also a general conclusion in this meta-analysis^24^. However, Tran et al. published in 2020 that there was no significant correlation between serum level of hemoglobin and neurological prognosis of IHCA patients^43^. Our heterogeneity and subgroup analysis contradict the findings of Tran et al., in that we found that high serum level of hemoglobin can improve neurological prognosis in OHCA, IHCA and OHCA + IHCA patients. Therefore, serum hemoglobin testing may help physicians and patients choose more appropriate treatments.

This meta-analysis has several limitations that should be noted. First, the number of included is small and subgroup analysis was prone to error. Second, some studies had high heterogeneity, and subgroup analysis indicated that cardiac arrest type and serum hemoglobin sampling time might be the sources of heterogeneity. All included studies were cohort studies (only two studies were multicenter studies). Thus, we need large multicentre prospective cohort type of study to confirm the results of this meta-analysis. To reduce heterogeneity, more data from other ethnic groups or countries should be included. Thirdly, the results may vary depending on the time point of CPC measurement^44^.

## Conclusions

The serum hemoglobin levels of patients who have survived after cardiac arrest may be associated with a better neurological prognosis, perhaps an appropriate increase in serum hemoglobin levels can improve the neurological prognosis of patients who have survived after cardiac arrest. It remains to be seen in future studies, what are the cut-off values of the serum hemoglobin levels that recommend the initiation of transfusion therapy to increase the chances of survival with a good neurological prognosis after cardiac arrest.

### Supplementary Information

Supplementary Information.

## Data Availability

The datasets used and/or analysed during the current study available from the corresponding author on reasonable request.
