# Supplementary material for: Hemoglobin as a prognostic marker for neurological outcomes in post-cardiac arrest patients: a meta-analysis
Source: Sci Rep. 2023 Oct 28;13:18531. doi: 10.1038/s41598-023-45818-5 (PMC10613227; doi:10.1038/s41598-023-45818-5)
Supplement: Supplementary file 1 — Supplementary Information. [file 41598_2023_45818_MOESM1_ESM.docx]

**Supplementary Table 1.** Comprehensive search strategy.

PubMed

| 1 | "Heart Arrest"[Mesh] | 55655 |
| --- | --- | --- |
| 2 | ((((((Arrest, Heart[Title/Abstract]) OR (Cardiac Arrest[Title/Abstract])) OR (Arrest, Cardiac[Title/Abstract])) OR (Asystole[Title/Abstract])) OR (Asystoles[Title/Abstract])) OR (Cardiopulmonary Arrest[Title/Abstract])) OR (Arrest, Cardiopulmonary[Title/Abstract]) | 47804 |
| 3 | ("Heart Arrest"[Mesh]) OR (((((((Arrest, Heart[Title/Abstract]) OR (Cardiac Arrest[Title/Abstract])) OR (Arrest, Cardiac[Title/Abstract])) OR (Asystole[Title/Abstract])) OR (Asystoles[Title/Abstract])) OR (Cardiopulmonary Arrest[Title/Abstract])) OR (Arrest, Cardiopulmonary[Title/Abstract])) | 76850 |
| 4 | "Death, Sudden, Cardiac"[Mesh] | 17860 |
| 5 | (((((((((Sudden Cardiac Death[Title/Abstract]) OR (Cardiac Death, Sudden[Title/Abstract])) OR (Death, Sudden Cardiac[Title/Abstract])) OR (Cardiac Sudden Death[Title/Abstract])) OR (Death, Cardiac Sudden[Title/Abstract])) OR (Sudden Death, Cardiac[Title/Abstract])) OR (Sudden Cardiac Arrest[Title/Abstract])) OR (Arrest, Sudden Cardiac[Title/Abstract])) OR (Cardiac Arrests, Sudden[Title/Abstract])) OR (Cardiac Arrest, Sudden[Title/Abstract]) | 27217 |
| 6 | ("Death, Sudden, Cardiac"[Mesh]) OR ((((((((((Sudden Cardiac Death[Title/Abstract]) OR (Cardiac Death, Sudden[Title/Abstract])) OR (Death, Sudden Cardiac[Title/Abstract])) OR (Cardiac Sudden Death[Title/Abstract])) OR (Death, Cardiac Sudden[Title/Abstract])) OR (Sudden Death, Cardiac[Title/Abstract])) OR (Sudden Cardiac Arrest[Title/Abstract])) OR (Arrest, Sudden Cardiac[Title/Abstract])) OR (Cardiac Arrests, Sudden[Title/Abstract])) OR (Cardiac Arrest, Sudden[Title/Abstract])) | 32457 |
| 7 | "Ventricular Fibrillation"[Mesh] | 17812 |
| 8 | ((Fibrillation, Ventricular[Title/Abstract]) OR (Fibrillations, Ventricular[Title/Abstract])) OR (Ventricular Fibrillations[Title/Abstract]) | 477 |
| 9 | ("Ventricular Fibrillation"[Mesh]) OR (((Fibrillation, Ventricular[Title/Abstract]) OR (Fibrillations, Ventricular[Title/Abstract])) OR (Ventricular Fibrillations[Title/Abstract])) | 18170 |
| 10 | "Tachycardia"[Mesh] | 51722 |
| 11 | ((Tachycardias[Title/Abstract]) OR (Tachyarrhythmia[Title/Abstract])) OR (Tachyarrhythmias[Title/Abstract]) | 15506 |
| 12 | ("Tachycardia"[Mesh]) OR (((Tachycardias[Title/Abstract]) OR (Tachyarrhythmia[Title/Abstract])) OR (Tachyarrhythmias[Title/Abstract])) | 58746 |
| 13 | (((("Heart Arrest"[Mesh]) OR (((((((Arrest, Heart[Title/Abstract]) OR (Cardiac Arrest[Title/Abstract])) OR (Arrest, Cardiac[Title/Abstract])) OR (Asystole[Title/Abstract])) OR (Asystoles[Title/Abstract])) OR (Cardiopulmonary Arrest[Title/Abstract])) OR (Arrest, Cardiopulmonary[Title/Abstract]))) OR (("Death, Sudden, Cardiac"[Mesh]) OR ((((((((((Sudden Cardiac Death[Title/Abstract]) OR (Cardiac Death, Sudden[Title/Abstract])) OR (Death, Sudden Cardiac[Title/Abstract])) OR (Cardiac Sudden Death[Title/Abstract])) OR (Death, Cardiac Sudden[Title/Abstract])) OR (Sudden Death, Cardiac[Title/Abstract])) OR (Sudden Cardiac Arrest[Title/Abstract])) OR (Arrest, Sudden Cardiac[Title/Abstract])) OR (Cardiac Arrests, Sudden[Title/Abstract])) OR (Cardiac Arrest, Sudden[Title/Abstract])))) OR (("Ventricular Fibrillation"[Mesh]) OR (((Fibrillation, Ventricular[Title/Abstract]) OR (Fibrillations, Ventricular[Title/Abstract])) OR (Ventricular Fibrillations[Title/Abstract])))) OR (("Tachycardia"[Mesh]) OR (((Tachycardias[Title/Abstract]) OR (Tachyarrhythmia[Title/Abstract])) OR (Tachyarrhythmias[Title/Abstract]))) | 148939 |
| 14 | "Hemoglobins"[Mesh] | 139583 |
| 15 | (((Hemoglobin[Title/Abstract]) OR (Eryhem[Title/Abstract])) OR (Ferrous Hemoglobin[Title/Abstract])) OR (Hemoglobin, Ferrous[Title/Abstract]) | 147648 |
| 16 | ("Hemoglobins"[Mesh]) OR ((((Hemoglobin[Title/Abstract]) OR (Eryhem[Title/Abstract])) OR (Ferrous Hemoglobin[Title/Abstract])) OR (Hemoglobin, Ferrous[Title/Abstract])) | 221553 |
| 17 | prognosis[MeSH:noexp] OR diagnosed[Title/Abstract] OR cohort*[Title/Abstract] OR cohort effect[MeSH Term] OR cohort studies[MeSH:noexp] OR predictor*[Title/Abstract] OR death[Title/Abstract] OR "models, statistical"[MeSH Term] | 3450196 |
| 18 | (((((("Heart Arrest"[Mesh]) OR (((((((Arrest, Heart[Title/Abstract]) OR (Cardiac Arrest[Title/Abstract])) OR (Arrest, Cardiac[Title/Abstract])) OR (Asystole[Title/Abstract])) OR (Asystoles[Title/Abstract])) OR (Cardiopulmonary Arrest[Title/Abstract])) OR (Arrest, Cardiopulmonary[Title/Abstract]))) OR (("Death, Sudden, Cardiac"[Mesh]) OR ((((((((((Sudden Cardiac Death[Title/Abstract]) OR (Cardiac Death, Sudden[Title/Abstract])) OR (Death, Sudden Cardiac[Title/Abstract])) OR (Cardiac Sudden Death[Title/Abstract])) OR (Death, Cardiac Sudden[Title/Abstract])) OR (Sudden Death, Cardiac[Title/Abstract])) OR (Sudden Cardiac Arrest[Title/Abstract])) OR (Arrest, Sudden Cardiac[Title/Abstract])) OR (Cardiac Arrests, Sudden[Title/Abstract])) OR (Cardiac Arrest, Sudden[Title/Abstract])))) OR (("Ventricular Fibrillation"[Mesh]) OR (((Fibrillation, Ventricular[Title/Abstract]) OR (Fibrillations, Ventricular[Title/Abstract])) OR (Ventricular Fibrillations[Title/Abstract])))) OR (("Tachycardia"[Mesh]) OR (((Tachycardias[Title/Abstract]) OR (Tachyarrhythmia[Title/Abstract])) OR (Tachyarrhythmias[Title/Abstract])))) AND (("Hemoglobins"[Mesh]) OR ((((Hemoglobin[Title/Abstract]) OR (Eryhem[Title/Abstract])) OR (Ferrous Hemoglobin[Title/Abstract])) OR (Hemoglobin, Ferrous[Title/Abstract])))) AND (prognosis[MeSH:noexp] OR diagnosed[Title/Abstract] OR cohort*[Title/Abstract] OR cohort[MeSH Terms] OR cohort studies[MeSH:noexp] OR predictor*[Title/Abstract] OR death[Title/Abstract] OR models, statistical[MeSH Term]) | 240 |

Web of Science

| 1 | ((((((((((((((TS=(heart arrest)) OR TI=(Arrest, Heart)) OR TI=(Cardiac Arrest)) OR TI=(Arrest, Cardiac)) OR TI=(Asystole)) OR TI=(Asystoles)) OR TI=(Cardiopulmonary Arrest)) OR TI=(Arrest, Cardiopulmonary)) OR AB=(Arrest, Heart)) OR AB=(Cardiac Arrest)) OR AB=(Arrest, Cardiac)) OR AB=(Asystole)) OR AB=(Asystoles)) OR AB=(Cardiopulmonary Arrest)) OR AB=(Arrest, Cardiopulmonary) |
| --- | --- |
| 2 | ((((((((((((((((((((TS=(Death, Sudden, Cardiac)) OR TI=(Sudden Cardiac Death)) OR TI=(Cardiac Death, Sudden)) OR TI=(Death, Sudden Cardiac)) OR TI=(Cardiac Sudden Death)) OR TI=(Death, Cardiac Sudden)) OR TI=(Sudden Death, Cardiac)) OR TI=(Sudden Cardiac Arrest)) OR TI=(Arrest, Sudden Cardiac)) OR TI=(Cardiac Arrests, Sudden)) OR TI=(Cardiac Arrest, Sudden)) OR AB=(Sudden Cardiac Death)) OR AB=(Cardiac Death, Sudden)) OR AB=(Death, Sudden Cardiac)) OR AB=(Cardiac Sudden Death)) OR AB=(Death, Cardiac Sudden)) OR AB=(Sudden Death, Cardiac)) OR AB=(Sudden Cardiac Arrest)) OR AB=(Arrest, Sudden Cardiac)) OR AB=(Cardiac Arrests, Sudden)) OR AB=(Cardiac Arrest, Sudden) |
| 3 | ((((((TS=(Ventricular Fibrillation)) OR TI=(Fibrillation, Ventricular)) OR TI=(Fibrillations, Ventricular)) OR TI=(Ventricular Fibrillations)) OR AB=(Fibrillation, Ventricular)) OR AB=(Fibrillations, Ventricular)) OR AB=(Ventricular Fibrillations) |
| 4 | ((((((TS=(Tachycardia)) OR TI=(Tachycardias)) OR TI=(Tachyarrhythmia)) OR TI=(Tachyarrhythmias)) OR AB=(Tachycardias)) OR AB=(Tachyarrhythmia)) OR AB=(Tachyarrhythmias) |
| 5 | #1 OR #2 OR #3 OR #4 |
| 6 | ((((((((TS=(Hemoglobins)) OR TI=(Hemoglobin)) OR TI=(Eryhem)) OR TI=(Ferrous Hemoglobin)) OR TI=(Hemoglobin, Ferrous)) OR AB=(Hemoglobin)) OR AB=(Eryhem)) OR AB=(Ferrous Hemoglobin)) OR AB=(Hemoglobin, Ferrous) |
| 7 | ((((((((((TS=(Prognosis)) OR TI=(Prognoses)) OR TI=(Prognostic Factors)) OR TI=(Factor, Prognostic)) OR TI=(Factors, Prognostic)) OR TI=(Prognostic Factor)) OR AB=(Prognoses)) OR AB=(Prognostic Factors)) OR AB=(Factor, Prognostic)) OR AB=(Factors, Prognostic)) OR AB=(Prognostic Factor) |
| 8 | #5 AND #6 AND #7 |

Cochrane

| #1 | MeSH descriptor: [Heart Arrest] explode all trees |
| --- | --- |
| #2 | (Arrest, Heart):ti,ab,kw OR (Cardiac Arrest):ti,ab,kw OR (Arrest, Cardiac):ti,ab,kw OR (Asystole):ti,ab,kw OR (Asystoles):ti,ab,kw |
| #3 | (Cardiopulmonary Arrest):ti,ab,kw OR (Arrest, Cardiopulmonary):ti,ab,kw |
| #4 | #1 or #2 or #3 |
| #5 | MeSH descriptor: [Death, Sudden, Cardiac] explode all trees |
| #6 | (Sudden Cardiac Death):ti,ab,kw OR (Cardiac Death, Sudden):ti,ab,kw OR (Death, Sudden Cardiac):ti,ab,kw OR (Cardiac Sudden Death):ti,ab,kw OR (Death, Cardiac Sudden):ti,ab,kw |
| #7 | (Sudden Death, Cardiac):ti,ab,kw OR (Sudden Cardiac Arrest):ti,ab,kw OR (Arrest, Sudden Cardiac):ti,ab,kw OR (Cardiac Arrests, Sudden):ti,ab,kw OR (Cardiac Arrest, Sudden):ti,ab,kw |
| #8 | #5 OR #6 OR #7 |
| #9 | MeSH descriptor: [Ventricular Fibrillation] explode all trees |
| #10 | (Fibrillation, Ventricular):ti,ab,kw OR (Fibrillations, Ventricular):ti,ab,kw OR (Ventricular Fibrillations):ti,ab,kw |
| #11 | #9 OR #10 |
| #12 | MeSH descriptor: [Tachycardia] explode all trees |
| #13 | (Tachycardias):ti,ab,kw OR (Tachyarrhythmia):ti,ab,kw OR (Tachyarrhythmias):ti,ab,kw |
| #14 | #12 OR #13 |
| #15 | #4 OR #8 OR #11 OR #14 |
| #16 | MeSH descriptor: [Hemoglobins] explode all trees |
| #17 | (Hemoglobins):ti,ab,kw OR (Eryhem):ti,ab,kw OR (Ferrous Hemoglobin):ti,ab,kw OR (Hemoglobin, Ferrous):ti,ab,kw |
| #18 | #5 or #6 |
| #19 | MeSH descriptor: [Prognosis] explode all trees |
| #20 | (Prognoses):ti,ab,kw OR (Prognostic Factors):ti,ab,kw OR (Prognostic Factor):ti,ab,kw OR (Factor, Prognostic):ti,ab,kw OR (Factors, Prognostic):ti,ab,kw |
| #21 | #19 OR #20 |
| #22 | #15 AND #18 AND #21 |

Embase

| #1 | 'heart arrest'/exp | 127289 |
| --- | --- | --- |
| #2 | 'arrest, heart':ab,ti OR 'cardiac arrest':ab,ti OR 'arrest, cardiac':ab,ti OR 'asystole':ab,ti OR 'asystoles':ab,ti OR 'cardiopulmonary arrest':ab,ti OR 'arrest, cardiopulmonary':ab,ti | 75459 |
| #3 | #1 OR #2 | 138614 |
| #4 | 'sudden cardiac death'/exp | 22745 |
| #5 | 'arrest, heart':ab,ti OR 'cardiac death, sudden':ab,ti OR 'death, sudden cardiac':ab,ti OR 'cardiac sudden death':ab,ti OR 'death, cardiac sudden':ab,ti OR 'sudden death, cardiac':ab,ti OR 'sudden cardiac arrest':ab,ti OR 'arrest, sudden cardiac':ab,ti OR 'cardiac arrests, sudden':ab,ti OR 'cardiac arrest, sudden':ab,ti | 5036 |
| #6 | #4 OR #5 | 25134 |
| #7 | 'heart ventricle fibrillation'/exp | 38999 |
| #8 | 'fibrillation, ventricular':ab,ti OR 'fibrillations, ventricular':ab,ti OR 'ventricular fibrillations':ab,ti | 722 |
| #9 | #7 OR #8 | 39364 |
| #10 | 'tachycardia'/exp | 185705 |
| #11 | 'fibrillation, ventricular':ab,ti OR 'fibrillations, ventricular':ab,ti OR 'ventricular fibrillations':ab,ti | 722 |
| #12 | #10 OR #11 | 186062 |
| #13 | #3 OR #6 OR #9 OR #12 | 323607 |
| #14 | 'hemoglobin'/exp | 456981 |
| #15 | 'hemoglobins':ab,ti OR 'eryhem':ab,ti OR 'ferrous hemoglobin':ab,ti OR 'hemoglobin, ferrous':ab,ti | 5577 |
| #16 | #14 OR #15 | 457582 |
| #17 | 'prognosis'/exp OR 'prognosis' | 1204019 |
| #18 | 'prognoses':ab,ti OR 'prognostic factors':ab,ti OR 'prognostic factor':ab,ti OR 'factor, prognostic':ab,ti OR 'factors, prognostic':ab,ti | 199443 |
| #19 | #17 OR #18 | 1262663 |
| #20 | #13 AND #16 AND #19 | 671 |

**Supplementary Table 2.** Baseline characteristics of individuals in the included studies

| **Author** | **Outcome** | **Sample size** | **OHCA, %** | **Age** | **Male, n (%)** | **CPR duration, min** | **TTM (%, Target temperature/duration)** † | **Hemoglobin(g/dL), median (IQR/SD)** |
| --- | --- | --- | --- | --- | --- | --- | --- | --- |
|  |  |  |  |  |  |  |  |  |
| SOS-KANTO study group | good | 34 | 100 | 55 (15) | 29 (85.3) | - | 9 (26.5) | 14.4 (1.6) |
|  | poor | 103 |  | 65 (22) | 65 (63.1) |  | 11 (10.7) | 12.8 (4.0) |
| Aiham Albaeni | good | 30 | 100 | 57(52–68) | 14(47) | 9.5 (4–13) | 17(57) | 12.5 (11.2–13.5) |
|  | poor | 116 |  | 66.5 (56–78) | 63(54) | 28.5 (16.5–40) | 54(47) | 10.8 (8.9–13.1) |
| K. Ameloot | good | 43 | - | 62 ± 13 | 35(81%) | - | all | 13.5 ± 1.7 |
|  | poor | 39 |  | 63 ± 13 | 22(56%) |  |  | 13.1 ± 2.0 |
| Federica ZAMA CAVICCHI | good | 157 | - | 58 [50-70] | 118 (75) | 11 [5-20] | 133 (85) | 13.0 [10.3-14.4] |
|  | poor | 257 | - | 65 [53-76] | 177 (69) | 18 [10-26] | 234 (91) | 11.5 [9.7-13.2] |
| Andrew Wormsbecker | good | 32 | - | N/SD 59.7(14.8) | 26 (81.3) | 10.0 (4.5–16.5) | - | 11.5 (20)(SD) |
|  | poor | 86 |  | 65.3 (14.7) | 68 (79.1) | 21.5(13.0–34.0) |  | 10.7 (19) |
| Chih-Hung Wang | good | 54 | 0 | 64.0 (15.0) | 43(79.6) | 9.0 (5.0–14.8) | - | 9.7 (3.1)SD |
|  | poor | 372 |  | 65.5 (16.7) | 224(60.2) | 14 (7–26) | - | 9.1 (2.4) |
| [Se Jong Oh](https://pubmed.ncbi.nlm.nih.gov/?term=Oh+SJ&cauthor_id=28807442) | survived | 164 | 100 |  | 75.0 | 26.5±12.6 | all | 14.3±2.2 |
|  | dead | 131 |  |  | 66.4 | 33.6±12.9 |  | 13.1±2.6 |
|  | good | 79 |  |  | 83.5 | 25.5±12.7 |  | 15.0±2.1 |
|  | poor | 216 |  |  | 66.7 | 21.0±9.2 |  | 13.3±2.4 |
| Young Mo Cho MD | good | 34 | 100 | 47(41.75-57.25) | 29 (85.3) | - | all | 15.70 (14.18-16.75) |
|  | poor | 83 |  | 53(42.00-63.00) | 53 (63.9) |  |  | 13.50 (11.40-14.90) |
| Kei Hayashida | good | 75 | 100 | 59 (14) | 58 (77.3) | - | 56 (74.7) | 13.9 (2.1) |
|  | poor | 420 |  | 69 (15) | 272 (64.8) |  | 131 (31.2) | 11.7 (2.2) |
| Adrien Bouglé | good | 22 | 100 | 58.7(15.9) | 17(77) | - | all | 13.1(1.8) |
|  | poor | 21 |  | 61.3(11.4) | 17(81) |  |  | 12.3(2.2) |
| Daesung Kim | good | 87 | 100 | 48(38-59) | 63(72.4) | 20(11-32) | all | 14.4(2.0) |
|  | poor | 159 |  | 57(44-70) | 105(66) | 34(24-48.5) |  | 12.8(2.5) |
| Abbreviations: OHCA = out-of-hospital cardiac arrest; CPR = cardiopulmonary resuscitation. | | | | | | | | |

**Supplementary Table 3.** Baseline characteristics of individuals in the included studies

| **Author** | **Outcome** | **Sample size** | **Non-VF/VT**  **(Asystole**  **And PEA)** | **Initial shockable rhythm**  **(VF/VT)n(%)** | **Collapse to chest compression** | **Time to ROSC**  **, min** | Bystander CPR  † |
| --- | --- | --- | --- | --- | --- | --- | --- |
|  |  |  |  |  |  |  |  |
| SOS-KANTO study group | good | 34 | - | 28 (82.4) | - | - | - |
|  | poor | 103 |  | 41 (39.8) |  |  |  |
| Aiham Albaeni | good | 30 | - | 13(40) | - | 9.5 (4–13)range | 9(30) |
|  | poor | 116 |  | 16(14) |  | 28.5 (16.5–40) | 37(32) |
| K. Ameloot | good | 43 | - | 34(79%) | - | - | 41(95%) |
|  | poor | 39 |  | 20(51%) |  |  | 25(65%) |
| Federica ZAMA CAVICCHI | good | 157 | 66(42) | - | - | 11 [5-20]IQR | 122 (78) |
|  | poor | 257 | 187(73) |  |  | 18 [10-26] | 155(60) |
| Andrew Wormsbecker | good | 32 | - | - | - | 10.0 (4.5–16.5)IQR | 30 (94.0) |
|  | poor | 86 |  |  |  | 21.5(13.0–34.0) | 73 (85.0) |
| Chih-Hung Wang | good | 54 | - | 21 (38.9) | - | 9.0 (5.0–14.8)IQR |  |
|  | poor | 372 |  | 76 (20.4) |  | 14 (7–26) |  |
| [Se Jong Oh](https://pubmed.ncbi.nlm.nih.gov/?term=Oh+SJ&cauthor_id=28807442) | good | 79 | 33(41.8) | (58.2) | - | 25.5±12.7 | 17.7 |
|  | poor | 216 | 172(79.5) | (20.6) |  | 31.2±13.1 | 7.4 |
| Young Mo Cho MD | good | 34 | 15(44.1) | 19 (55.9) | 5 (2-10) | 24.00 IQR(15.00-37.00) | 10 (29.4) |
|  | poor | 83 | 67(80.7) | 16 (19.3) | 7 (4-12) | 38.00 (24.00-51.00) | 10 (12.0) |
| Kei Hayashida | good | 75 | - | 43 (57.3) | - |  | 47 (62.7) |
|  | poor | 420 |  | 78 (18.6) |  |  | 152 (36.2) |
| Adrien Bouglé | good | 22 | - | 18(82) | - | - | 22(100) |
|  | poor | 21 |  | 17(81) |  |  | 19(90) |
| Daesung Kim | good | 87 | - | 64 (73.6) | - | 20(11-32)IQR | 57 (65.5) |
|  | poor | 159 |  | 30 (18.9) |  | 34(24-48.5) | 84 (52.8) |
| VF=ventricular fibrillation/, VT= pulseless ventricular tachycardia, PEA=pulseless electrical activity, ROSC=return of spontaneous circulation, CPR=cardiopulmonary resuscitation. | | | | | | | |


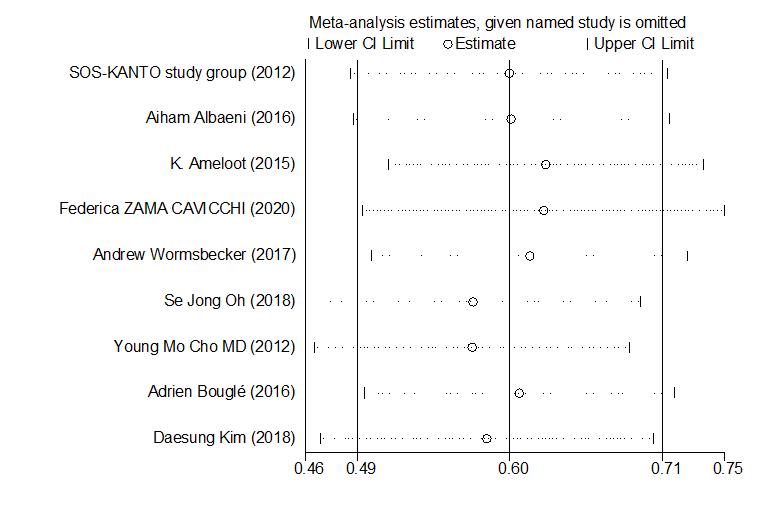


**Supplementary Fig. 1.** Sensitivity analysis


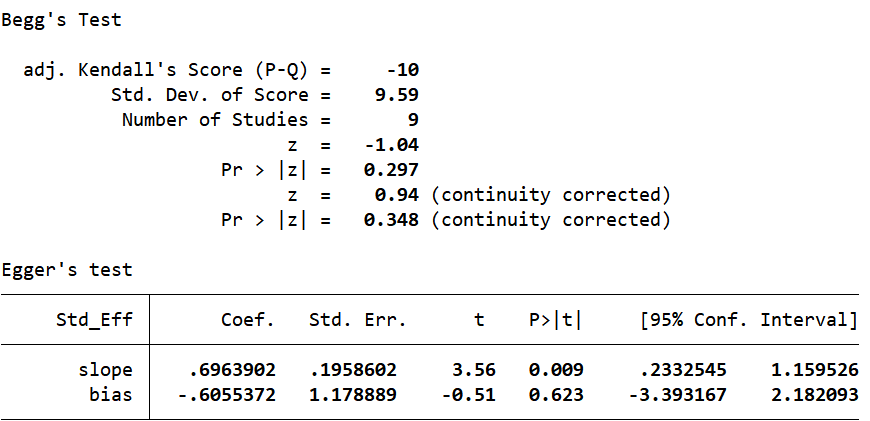


**Supplementary Fig. 2.** Begg^’^s test and Egger^’^s test


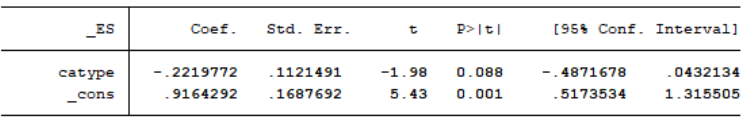


**Supplementary Fig. 3.** Regression analysis


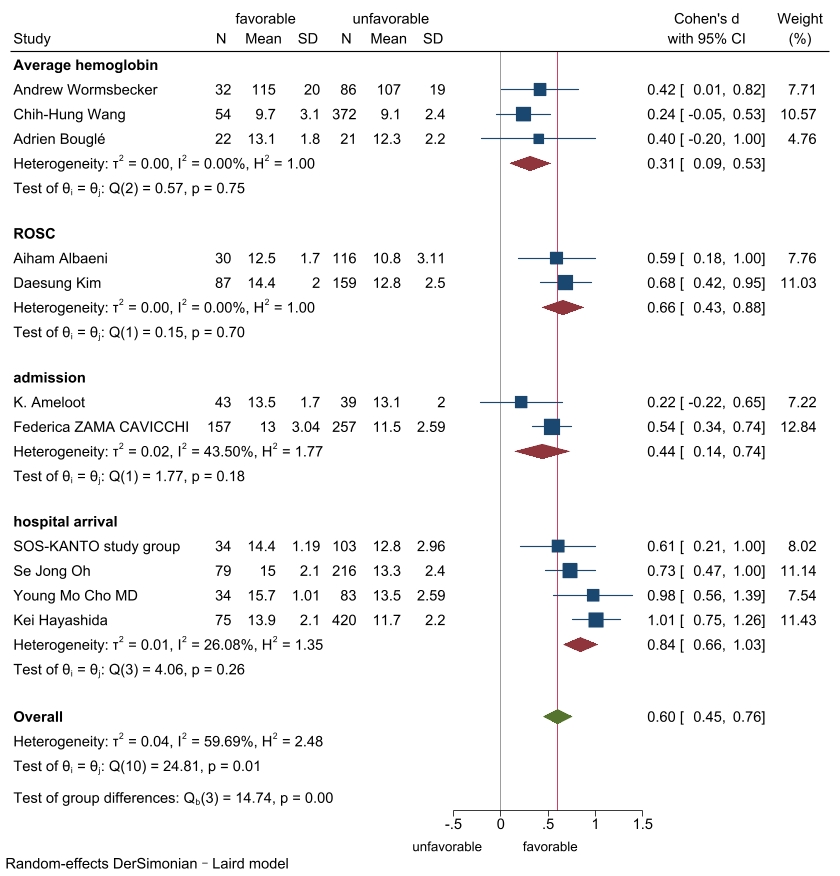


**Supplementary Fig. 4.** favorable: good neurological prognosis group; unfavorable: poor neurological prognosis group. ROSC: return of spontaneous circulation.


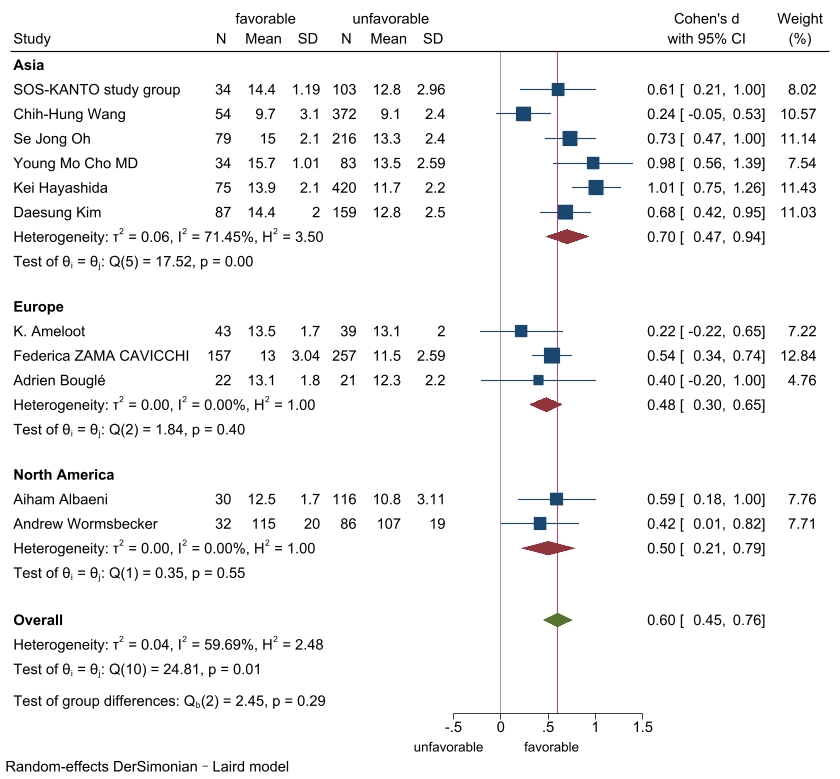


**Supplementary Fig. 5. favorable**: good neurological prognosis group; unfavorable: poor neurological prognosis group.


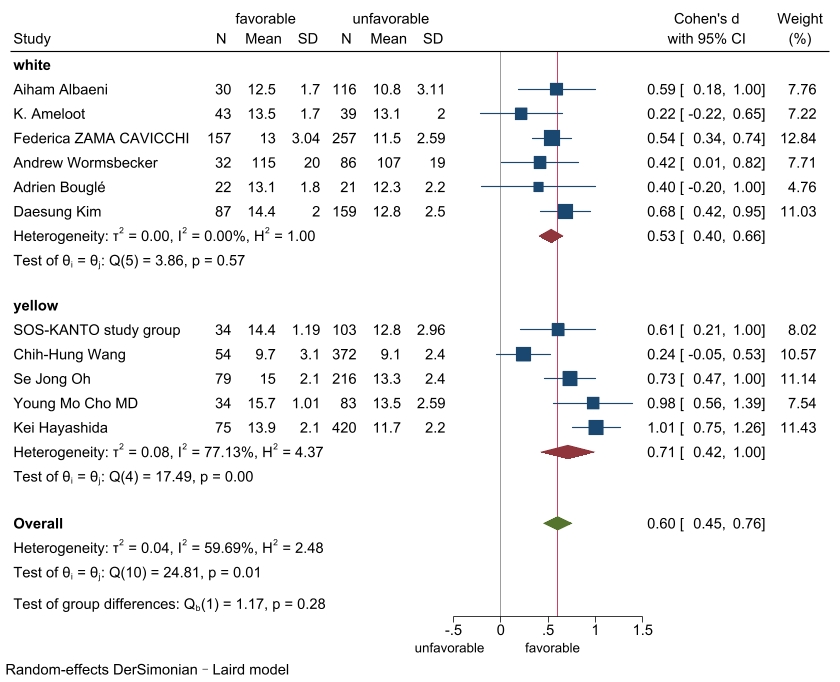


**Supplementary Fig. 6.** favorable: good neurological prognosis group; unfavorable: poor neurological prognosis group.


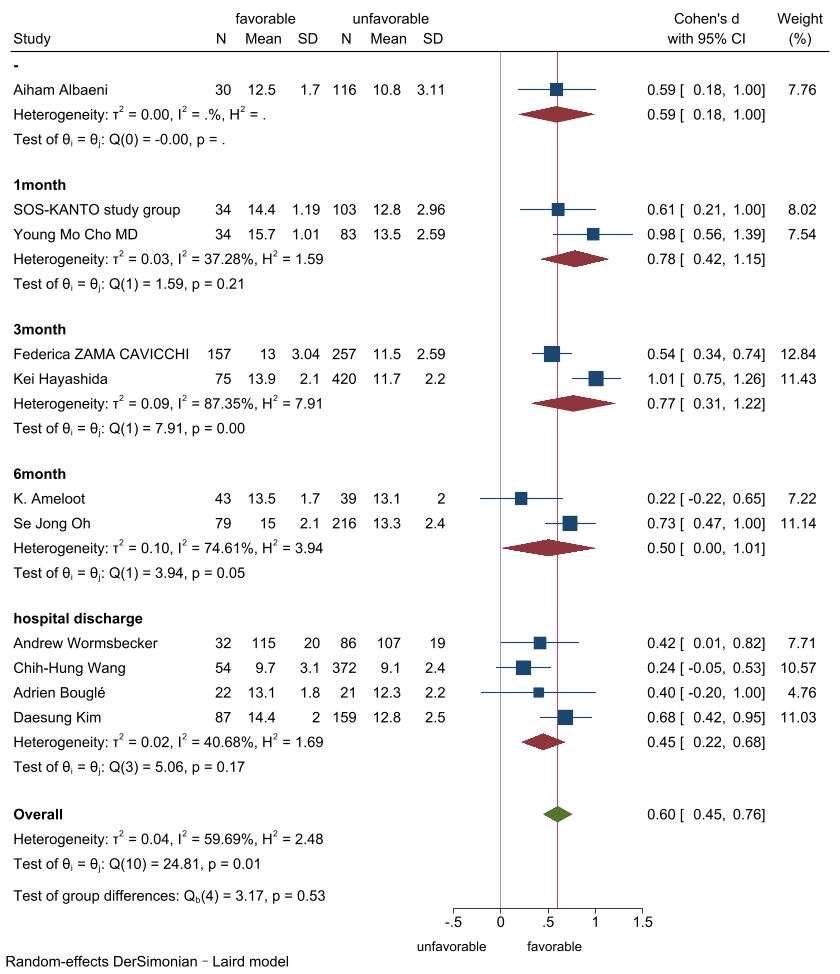


**Supplementary Fig. 7.** favorable: good neurological prognosis group; unfavorable: poor neurological prognosis group.

**
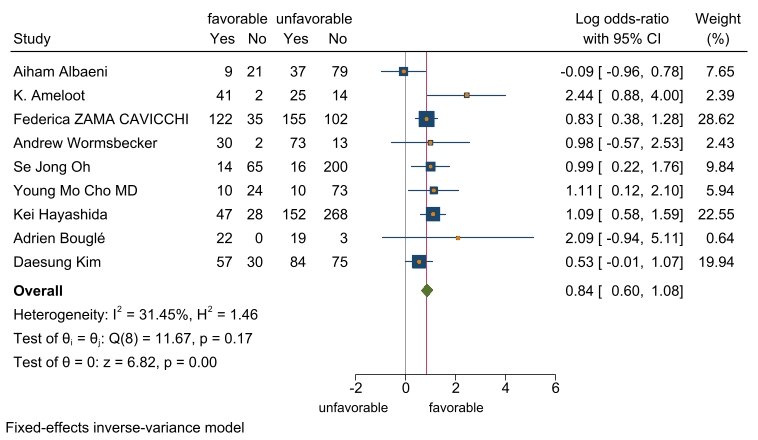
**

**Supplementary Fig. 8.** Relationship between bystander CPR and neurological outcomes. favorable: good neurological prognosis group; unfavorable: poor neurological prognosis group.

**
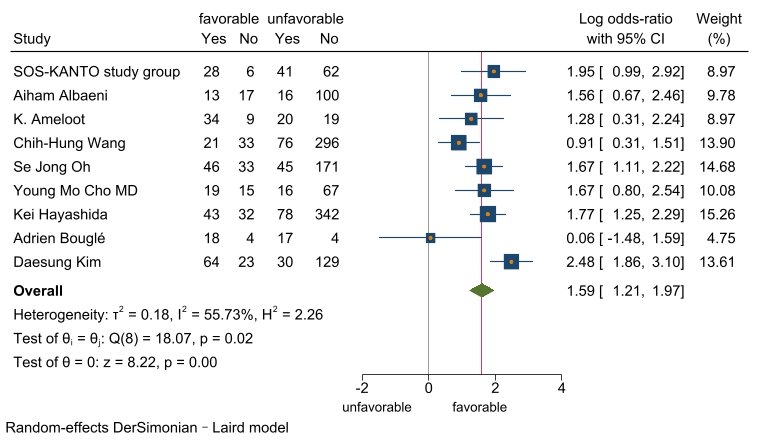
**

**Supplementary Fig. 9.** Relationship between initial shockable rhythm and neurological outcomes. favorable: good neurological prognosis group; unfavorable: poor neurological prognosis group.

**
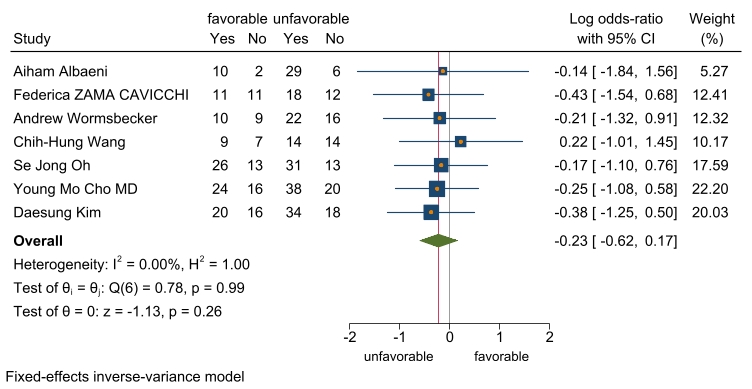
**

**Supplementary Fig. 10.** Relationship between time to ROSC and neurological outcomes. favorable: good neurological prognosis group; unfavorable: poor neurological prognosis group.

**
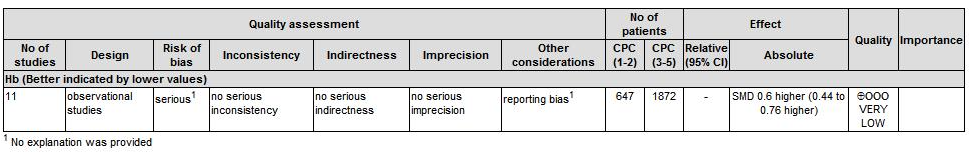
**

**Supplementary Fig. 11.** GRADE profile

**
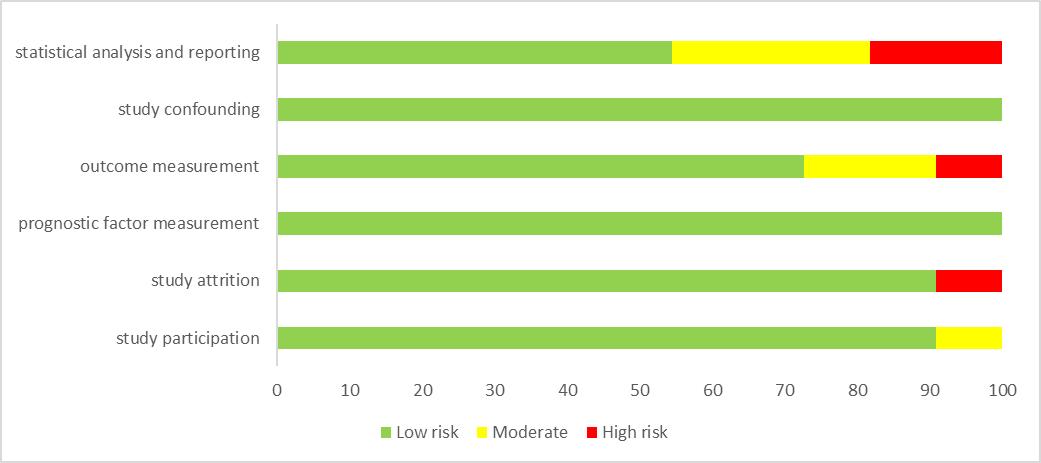
**

**Supplementary Fig. 12.** To assess the risk of bias, according to the Quality in Prognostic Studies (QUIPS) tool.
